# Erythroid Differentiation Regulator 1 as a Regulator of Neuronal GSH Synthesis

**DOI:** 10.3390/antiox13070771

**Published:** 2024-06-26

**Authors:** Wattanaporn Bhadhprasit, Chisato Kinoshita, Nobuko Matsumura, Koji Aoyama

**Affiliations:** Department of Pharmacology, Teikyo University School of Medicine, Tokyo 173-8605, Japan; sumidaw@med.teikyo-u.ac.jp (W.B.); ciitaka@med.teikyo-u.ac.jp (C.K.); nmatsumu@med.teikyo-u.ac.jp (N.M.)

**Keywords:** glutamate transporter-associated protein 3-18 (GTRAP3-18), excitatory amino acid carrier 1 (EAAC1), oxidative stress

## Abstract

Erythroid differentiation regulator 1 (Erdr1) is a cytokine known to play important roles in cell survival under stressful conditions, maintenance of cellular growth homeostasis, and activation of the immune system. However, the impact of Erdr1 on neurons remains undefined. In this study, we present novel evidence that Erdr1 plays a role in regulating glutathione (GSH) synthesis via glutamate transporter-associated protein 3-18 (GTRAP3-18), an anchor protein in the endoplasmic reticulum that holds excitatory amino acid carrier 1 (EAAC1) in neurons. Both DNA microarray and quantitative real-time PCR analyses revealed an approximately 2-fold increase in Erdr1 levels in the hippocampus of GTRAP3-18-deficient mice compared to those of wild-type mice. Knockdown of Erdr1 *in vitro* resulted in a decrease in GTRAP3-18 levels, leading to an increase in EAAC1 expression and intracellular GSH levels, and subsequently, cytoprotective effects against oxidative stress. Our findings shed light on the regulatory mechanisms involving Erdr1, GTRAP3-18, EAAC1, and GSH in the context of neuronal defense against oxidative stress. Understanding the intricate interplay among these molecules may pave the way for the development of promising therapeutic strategies for neurodegenerative disorders.

## 1. Introduction

Oxidative stress has been proposed to contribute to the dysfunction or demise of neuronal cells in individuals with neurodegenerative diseases [1,2]. The accrual of surplus oxidants, in conjunction with the insufficient functionality of antioxidant defense mechanisms, results in a perturbation of homeostasis in the equilibrium between pro-oxidants and antioxidants. This imbalance precipitates the heightened generation of reactive oxygen species (ROS). The extensive damage caused by ROS to lipids, proteins, and nucleic acids ultimately leads to cell death [3]. It is noteworthy that neurons are particularly vulnerable to oxidative damage due to their high content of polyunsaturated fatty acids, which are targets of lipid peroxidation, along with high oxygen consumption and relatively low levels of antioxidants [3]. Elevated levels of ROS have been implicated in various neurodegenerative diseases such as Alzheimer’s disease (AD), Parkinson’s disease (PD), Huntington’s disease, and amyotrophic lateral sclerosis [4,5,6,7,8,9]. Given the pivotal role of oxidative stress in neurodegenerative diseases, any strategy capable of neutralizing ROS may hold promise for preventing or slowing down the progression of neurodegeneration.

Glutathione (GSH), a prominent antioxidant in the brain [10], plays critical roles in the antioxidant defense system, including acting as a direct antioxidant, an enzyme cofactor, and a cysteine storage form and maintaining neuronal redox homeostasis [3]. GSH is a principal intracellular thiol compound composed of glutamate, glycine, and cysteine, with the latter serving as a crucial substrate for GSH synthesis in neurons [3,10,11]. Neuronal cysteine uptake is predominantly mediated by excitatory amino acid carrier 1 (EAAC1), also known as excitatory amino acid transporter (EAAT) type 3 [3]. Dysfunction of EAAC1 leads to impaired neuronal GSH synthesis and metabolism, heightened oxidative stress, and age-dependent neurodegeneration [12]. EAAC1 expression is negatively regulated by glutamate transporter-associated protein 3-18 (GTRAP3-18), an endoplasmic reticulum (ER) protein belonging to the prenylated Rab acceptor family [13]. GTRAP3-18 binds to EAAC1 in neurons and retains it within the ER, thereby inhibiting EAAC1-mediated cysteine uptake on the plasma membrane [3,14]. Reducing GTRAP3-18 expression in neurons significantly enhances neuroprotective activity and confers resistance against neurodegeneration by promoting EAAC1-mediated cysteine uptake, thereby increasing neuronal GSH synthesis [15]. Inhibiting GTRAP3-18 could be a promising strategy to enhance neuronal GSH levels, thereby offering neuroprotection against ROS in the brain.

Erythroid differentiation regulator 1 (Erdr1), initially identified as a hemoglobin synthesis factor in human and murine erythroleukemia cell lines [16], is a cytokine widely expressed in various normal mouse and human tissues [17]. Erdr1 plays an essential role in maintaining cellular growth homeostasis under stressful conditions [17]. Recent evidence has also unveiled additional functions of Erdr1 in cancer and skin diseases [18,19,20]. However, its function in the central nervous system (CNS) remains unclear. Although Erdr1 is known to act as a stress-related survival factor [18,20], its potential role in neuronal defense against oxidative stress has not been explored. Furthermore, there is currently no information on the relationship between Erdr1 and GSH synthesis in neurons. In the present study, we examined gene expression patterns in the hippocampus of GTRAP3-18-deficient mice using DNA microarray analysis and found a significant increase in *Erdr1* expression. We further investigated the relationship between GTRAP3-18 and Erdr1, focusing on GSH synthesis, using the mouse neuroblastoma cell line Neuro2a and primary mouse hippocampal neurons. Knockdown of Erdr1 *in vitro* significantly downregulates GTRAP3-18 expression, leading to an increase in EAAC1 expression and a subsequent elevation of GSH levels, which have been associated with neuronal defense against oxidative stress. These findings suggest that Erdr1 plays a key role in regulating neuronal GSH synthesis via the EAAC1/GTRAP3-18 pathway. This study provides a comprehensive analysis of the interplay between Erdr1 and GTRAP3-18, highlighting its potential as a therapeutic target for enhancing neuronal defense against oxidative stress.

## 2. Materials and Methods

### 2.1. Animals

Eight-week-old male mice lacking GTRAP3-18 (GTRAP3-18 KO) and wild-type mice were used in the experiments; these lines were described previously [15]. All mice were housed in a temperature-controlled, pathogen-free room at 23 °C under a 12-h light/dark cycle with food and water available *ad libitum*. All animal procedures were performed according to a research protocol approved by the Teikyo University Animal Ethics Committee.

### 2.2. Cell Culture

Neuro2a cells were purchased from KAC (Kyoto, Japan). The cells were grown in MEM (Nacalai Tesque, Kyoto, Japan) supplemented with 10% fetal bovine serum (Gibco, Waltham, MA, USA), non-essential amino acids (Wako, Osaka, Japan), and 1% penicillin–streptomycin (Gibco) at 37 °C in a 5% CO_2_ humidified incubator (Panasonic, Osaka, Japan). The cultured cells were passaged every 2 to 3 days at 1 × 10^5^ to 3 × 10^5^ cells per 100-mm dish.

Primary mouse hippocampal neurons were purchased from Thermo Fisher Scientific (Waltham, MA, USA). The cells were plated onto poly-D-lysine-coated (4.5 μg/cm^2^) coverslips, with cell densities at 50,000/well, in a 24-well plate and incubated in a Neurobasal^TM^ medium, supplemented with B-27^TM^ (Thermo Fisher Scientific) at 37 °C in a humidified atmosphere of 5% CO_2_ in air. After 24 h of incubation, half of the medium was removed and replaced with fresh medium. The cells were fed by replacing half the volume with fresh medium every third day.

### 2.3. Transfection

Neuro2a cells were transfected with an siRNA specific for Erdr1 and a scrambled siRNA as its negative control (Ambion, Austin, TX, USA) using Lipofectamine^®^ RNAiMax (Invitrogen, Waltham, MA, USA) according to the manufacturer’s protocol as follows: An siRNA specific for Erdr1 (25 pmol) or scrambled siRNA (25 pmol) was diluted in 250 μL of MEM supplemented with non-essential amino acids (Wako), 1% penicillin–streptomycin (Gibco) without serum and 7.5 μL of Lipofectamine^®^ RNAiMAX in a single well of a 6-well plate. The siRNA–lipid complex solution was added to 0.25–1 × 10^6^ cells/well. Transfection was continued for 24 h and then the medium was exchanged for fresh medium. After a further 24 h of incubation, quantitative real-time PCR, Western blotting, immunocytochemical analysis of EAAC1 expression, GSH production, intracellular ROS measurements, and cytotoxicity assay were performed.

Primary mouse hippocampal neurons at 7 days in vitro (DIV) were transfected with an siRNA specific for Erdr1, labeled with Cy^®^3 dye, or a scrambled siRNA, also labeled with Cy^®^3 dye, as a negative control (Ambion). The transfection was carried out using magnetofection with the NeuroMag^TM^ transfection reagent (OZ Biosciences, San Diego, CA, USA) following the manufacturer’s protocol. An siRNA specific for Erdr1, labeled with Cy^®^3 dye (50 nM), and a scrambled siRNA, also labeled with Cy^®^3 dye (50 nM), were diluted in 50 μL of B-27^TM^ supplement-free Neurobasal^TM^ medium. To this solution, 2 μL of NeuroMag reagent was added, and the resulting complex solution was then applied to the primary neurons. The cell culture plate was subsequently positioned on the magnetic plate for a duration of 30 min. The transfection medium was changed right after the magnetofection procedure with a fresh, pre-warmed Neurobasal^TM^ medium supplemented with B-27^TM^. After a further 24 h of incubation, transfected primary hippocampal neurons were washed twice with phosphate buffer saline (PBS), and then fixed with 4% paraformaldehyde (PFA). The cells were permeabilized with 0.05%Triton-X100. After a wash with PBS, the cells were mounted using Fluoromount-Plus mounting solution (Diagnostic Biosystems, Pleasanton, CA, USA) and captured with a KEYENCE BZ-X800 fluorescence microscope (KEYENCE Corporation, Osaka, Japan).

Primary mouse hippocampal neurons at DIV7 were transfected with an siRNA specific for Erdr1 and a scrambled siRNA (Ambion) using the magnetofection transfection reagent NeuroMag^TM^ (OZ Biosciences), following the manufacturer’s protocol as described above. After an additional 24 h of incubation, transfected primary hippocampal neurons were captured with an Olympus CKX53 (Olympus Corporation, Tokyo, Japan) and then exposed to 0.5 μM of CellTracker^TM^ green 5-chloromethylfluorescein diacetate (CMFDA) for 30 min in B-27^TM^ supplement-free Neurobasal^TM^ medium. Subsequently, the CMFDA-labeled cells were fixed with 4% PFA in PBS for 10 min, and the CMFDA signal intensity was measured using a Zeiss LSM 880 confocal laser scanning microscope (Carl Zeiss, Oberkochen, Germany). The fluorescence intensity of labeled cells was quantified using Fiji (ImageJ-win64), an open-source platform for biological image analysis.

### 2.4. DNA Microarray

Total RNA was extracted from the hippocampus of GTRAP3-18 KO and wild-type mice using Trizol Reagent (Life Technologies, Carlsbad, CA, USA) according to the manufacturer’s protocol. RNA samples were analyzed with a GeneChip^TM^ Mouse Gene 2.0 ST Array (Thermo Fisher Scientific) by Filgen. The samples were scanned by a GeneChip^TM^ Scanner 3000 7G (Thermo Fisher Scientific) and normalized by robust multichip average (RMA) normalization. Normalization data are shown as the ratio of the expression of the GTRAP3-18 KO mice gene to that of the wild-type.

### 2.5. Quantitative Real-Time PCR

Total RNA was prepared using Trizol Reagent (Life Technologies) according to the manufacturer’s protocol. Quantitative real-time PCR was performed using a ReverTra Ace^®^ qPCR RT Kit (Toyobo, Osaka, Japan) in combination with a PrimePCR Probe Assay specific for mouse Erdr1 (BioRad, Hercules, CA, USA) or SYBR Green reagent (Thermo Fisher Scientific). Erdr1 mRNA was quantified using a PrimePCR Probe Assay for Erdr1. GTRAP3-18 and GAPDH mRNAs were detected with Fast SYBR^®^ Green Master Mix and a gene-specific primer set. The forward and reverse primers for GTRAP3-18 were 5′-GGAACAACCGTGTAGTGAGCAA-3′ and 5′-TGATGCCGAACACAAAGACC-3′, respectively. The forward and reverse primers for GAPDH were 5′-AAAATGGTGAAGGTCGGTGTG-3′ and 5′-AATGAAGGGGTCGTTGATGG-3′, respectively. Quantitative real-time PCRs were performed using an Applied Biosystem 7500 Fast Real-Time PCR System (Thermo Fisher Scientific) under the following cycling conditions: For Erdr1, initial denaturation at 95 °C for 2 min, 50 cycles of denaturation at 95 °C for 5 s, and annealing and extension at 60 °C for 30 s were used. For GTRAP3-18 and GAPDH, enzyme activation at 95 °C for 20 s, 45 cycles of denaturation at 95 °C for 3 s, and annealing and extension at 60 °C for 30 s were used. To confirm the target specificity, dissociation curve analysis was performed at the end of the run that used the SYBR Green reagent.

### 2.6. Western Blotting

Either whole brain samples or hippocampus samples were homogenized using RIPA buffer (50 mM Tris-HCl pH 7.2/150 mM NaCl/1% NP-40/0.25% sodium deoxycholate/1 mM EDTA/1 mM PMSF/1 mM NaF/1 mM Na_3_Vo_4_/5 μg/mL of leupeptin, pepstatin, and aprotinin) on ice. Neuro2a cells were washed with ice-cold PBS, scraped into RIPA buffer, and homogenized under rotary agitation for 30 min at 4 °C. After centrifugation at 15,000 rpm for 15 min, the supernatant solutions of whole brain samples, hippocampus samples or Neuro2a cells were analyzed with a Pierce^TM^ BCA Protein Assay Kit (Thermo Fisher Scientific) to determine the protein amounts. The protein samples were mixed (1:1) with 2×Laemmli buffer (125 mM Tris/HCl pH 6.8/20% glycerol/4% SDS/10% β-mercaptoethanol/2% dithiothreitol/0.004% bromphenol blue in ethanol) and boiled for 3 min before use. Equal amounts of total cellular protein were separated by sodium dodecyl sulfate-polyacrylamide gel electrophoresis and transferred to polyvinylidene fluoride (PVDF) membranes (Bio-Rad). Non-specific binding was blocked with 5% skim milk in 0.01% bovine serum albumin (BSA)/0.5% Tween-20/0.05% sodium azide/10 mM Tris-buffered saline, pH 7.4, and membranes were incubated overnight at 4 °C with anti-mouse Arl6ip5 (GTRAP3-18) polyclonal antibody (Abnova, Taipei, Taiwan) at 1:1000 dilution, anti-mouse Erdr1 polyclonal antibody (Antibody Research Corporation, St Charles, Missouri, USA) at 1:1000 dilution, anti-EAAC1 antibody (Allomone labs, Jerusalem, Israel) at 1:1000 dilution, and anti-beta actin monoclonal antibody (Thermo Fisher Scientific) at 1:5000 dilution. After washing, the membranes were incubated with horseradish peroxidase-conjugated anti-rabbit IgG antibody (MP Biochemicals, Santa Ana, CA, USA) at 1:5000 dilution or anti-mouse IgG antibody (Merck, Darmstadt, Germany) at 1:10,000 dilution and detected with Amersham ECL Prime Western Blotting Detection Reagent (Cytiva, Marlborough, MA, USA). Immunoblotting data were collected using a Luminograph I (ATTO) measuring emitted photons by chemiluminescence. Protein expression was then evaluated using CS Analyzer 4 software (ver. 2.2.3; ATTO, Tokyo, Japan).

### 2.7. Immunocytochemistry

To determine EAAC1 expression in Neuro2a cells, Neuro2a cells transfected with an siRNA specific for Erdr1 or with a scrambled siRNA were fixed with 4% PFA and then permeabilized with 0.05% Triton-X100. Non-specific staining was blocked with the reagent PBS containing 5% BSA, and the cells were incubated with anti-EAAC1 (Alomone Labs) at 1:1000 dilution for 1 h. After a wash with PBS-Tween20, the cells were labeled with fluorescent-labeled secondary antibodies Alexa-Fluor 488 anti-rabbit IgG (Invitrogen) at 1:1000 dilutions for 30 min. Finally, the cells were mounted using a Fluoromount-Plus mounting solution (Diagnostic Biosystems) and captured with a KEYENCE BZ-X800 fluorescence microscope (KEYENCE Corporation). The fluorescence intensity of the labeled cells was quantified by Fiji.

### 2.8. Fluorescence Microscopic Analysis of GSH Production in Neuro2a Cells

To analyze cellular GSH production in Neuro2a, Neuro2a cells transfected with an siRNA specific for Erdr1 or with a scrambled siRNA were incubated in 10 μM of CellTracker^TM^ Green CMFDA (Thermo Fisher Scientific) for 30 min in the serum-free MEM (Nacalai Tesque) supplemented with non-essential amino acids (Wako) and 1% penicillin–streptomycin (Gibco). The CMFDA-labeled cells were fixed with 4% PFA in PBS for 10 min and then the CMFDA signal intensity was measured with a Nikon A1 confocal microscope (Nikon Corporation, Tokyo, Japan). The fluorescence intensity of labeled cells was quantified by Fiji.

### 2.9. Extraction of GSH from Neuro2a Cells and Measurement of GSH Levels

Neuro2a cells transfected with siRNA specific for Erdr1 or with scrambled siRNA were washed with ice-cold PBS and scraped into 0.1 M perchloric acid. Each sample was centrifuged at 20,000× *g* for 15 min at 4 °C. Supernatants were used for detection of GSH levels. GSH levels in the cultured Erdr1 siRNA-transfected or scrambled siRNA-transfected Neuro2a cells were determined using a high-performance liquid chromatography (HPLC) system with 4-Fluoro-7-sulfamoylbenzofurazan (ABD-F), a fluorogenic labeling reagent for thiols. Before the labeling, the pH of each sample was adjusted to 5.0–5.6 with neutralizing buffer containing 0.1 M NaOH/0.1 M CH_3_COONa. The samples were then incubated with 0.5 mM ABD-F in a borate buffer at 50 °C for 5 min. The reactions were stopped by cooling on ice and adding HCl. Fifty microliter aliquots of the reaction samples were injected into the reverse-phase HPLC column. GSH detection was performed using a Nexera X2 Ultra HPLC system (Shimadzu, Kyoto, Japan) consisting of a communications bus module (CBM-20A), liquid chromatograph (LC30AD), auto sample (SIL-30AC), fluorescence detector (RF-20Axs), and column oven (CTO-20AC). An Inertsil ODS-2 analytical column (150 mm × 4.6 mm ID 5 μm) (GL Sciences, Tokyo, Japan) was fixed at 40 °C and connected to a corresponding guard column (10 mm × 4.0 mm ID 5 μm; GL Sciences). A stepwise gradient elution was programmed with solvent A (50 mM potassium biphthalate at pH 4.0) and solvent B (8% acetonitrile in solvent A). The mobile phase was held at 80% solvent A and 20% solvent B for 6 min, followed by a 10 min program held at 100% solvent B. The flow rate of the eluate was 1.0 mL/min. All samples were injected into the column with an auto sampler. ABD-F-labeled GSH was detected by the fluorescence detector with excitation at 380 nm and emission at 510 nm. The signals from the detector were analyzed by LabSolutions (Shimadzu). The GSH concentrations were calculated from the peak area standardized with known amounts of GSH.

### 2.10. Intracellular ROS Measurements

Neuro2a cells were seeded in a 96-well plate at a density of 3 × 10^4^ cells/well. The cells were transfected with either an siRNA specific for Erdr1 or a scrambled siRNA using Lipofectamine^®^ RNAiMax (Invitrogen). Intracellular ROS levels in Neuro2a cells were assessed using ROS Assay Kit-Highly Sensitive DCFH-DA (Dojindo, Kumamoto, Japan) following the manufacturer’s protocol. The culture medium was discarded, and the cells were gently washed twice with PBS. One-hundred microliters of a diluted, highly sensitive 2′, 7′-dichlorofluorescin diacetate (DCFH-DA) dye solution (1:1000) was added, and then the plate was incubated at 37 °C for 30 min. After removing the dye solution, the cells were washed twice with PBS and then treated with 100 μM H_2_O_2_ for 30 min. The oxidation of DCFH by ROS leads to a fluorescent derivative 2′, 7′-dichlorofluorescin (DCF), whose fluorescence is proportional to the total ROS levels in the sample. The H_2_O_2_ treated cells were washed twice with PBS, and the fluorescence signals were measured at 485 nm excitation/535 nm emission using a DTX 800 Multimode Detector (Beckman Coulter, Brea, CA, USA).

### 2.11. Cytotoxicity Assay

Neuro2a cells were seeded in a 96-well plate at a density of 4 × 10^4^ cells/well. The cells were transfected with an siRNA specific for Erdr1 or with scrambled siRNA using Lipofectamine^®^ RNAiMax (Invitrogen) according to the manufacturer’s protocol. After a further 24 h of incubation in the presence of 100 μM H_2_O_2_, neuroprotective activity was quantified and compared between the cells transfected with Erdr1-specific siRNA and those transfected with scrambled siRNA by measuring the lactate dehydrogenase (LDH) released into the cell culture medium in response to plasma membrane damage using an LDH Assay Kit-WST (Dojindo).

### 2.12. Statistical Analysis

Data are expressed as the mean ± standard error of the mean (SEM) obtained from independent samples. The Mann–Whitney *U* test was used for statistical analysis. Differences with *p* < 0.05 were considered statistically significant.

## 3. Results

### 3.1. Erdr1 Expression in the Whole Brain and Hippocampus of GTRAP3-18-Deficient Mice

**Figure 1:**
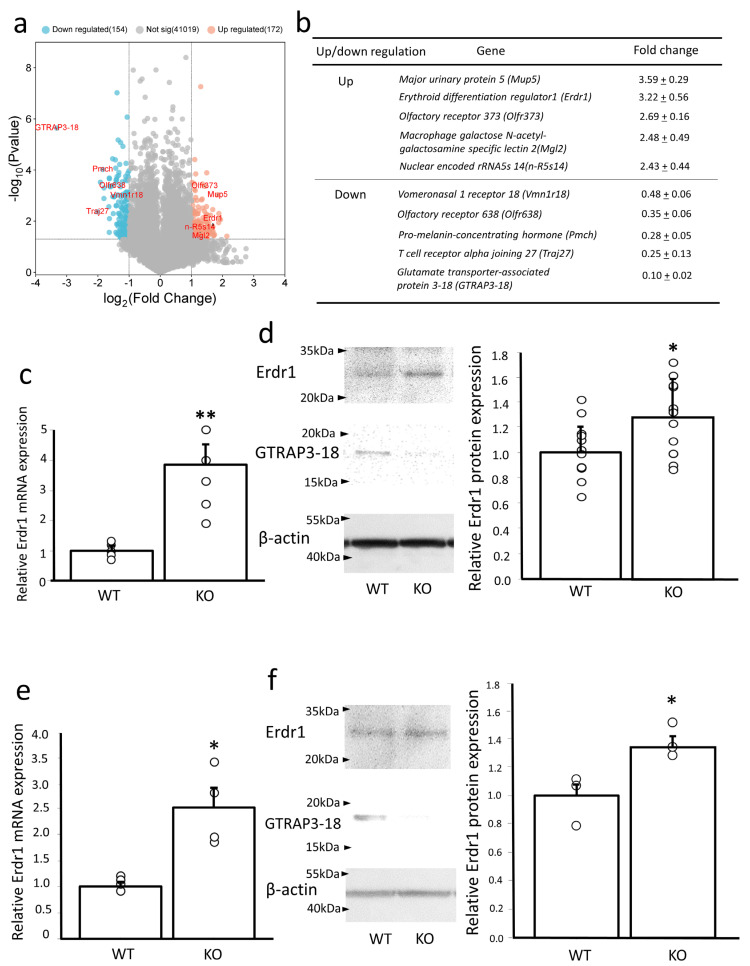
Erdr1 expression was upregulated in the brain and the hippocampus of GTRAP3-18-deficient mice. (**a**) Volcano plot of differential gene expression. The volcano plot shows the fold change (*x*-axis) versus the significance (*y*-axis) of 41,345 genes. The fold change and the significance (*p*-value) are converted to log_2_(fold change) and −log_10_(*p*-value), respectively. The vertical and horizontal gray lines show the cut-off of fold change ±2.0, and *p*-value 0.05, respectively. Single genes are depicted as dots. The expression levels of 172 genes were significantly upregulated (in red) and 154 genes were significantly downregulated (in blue). (**b**) Microarray gene expression analysis in the hippocampus. Up-regulation (≥2-fold changes) and down-regulation (≤0.5-fold changes) of GTRAP3-18-deficient mice genes are presented as the fold differences compared to those of the wild-type. Values are presented as mean of fold change ± standard error of the mean (SEM); n = 3. (**c**) Quantitative real-time PCR analysis of Erdr1 in the hippocampus of GTRAP3-18-deficient mice (KO) and wild-type mice (WT). Data are shown as the ratio of Erdr1 to GAPDH mRNA expression. n = 5. ** *p* < 0.01. (**d**) Western blotting analysis of Erdr1 in the hippocampus of GTRAP3-18-deficient mice (KO) and wild-type mice (WT). Quantification of the Erdr1 protein expression (data in the left panel) by densitometry is shown in the right panel. n = 13. * *p* < 0.05. (**e**) Quantitative real-time PCR analysis of Erdr1 in the brains of GTRAP3-18-deficient mice (KO) and wild-type mice (WT). Data are shown as the ratio of Erdr1 to GAPDH mRNA expression. n = 4. * *p* < 0.05. (**f**) Western blotting analysis of Erdr1 in the brains of GTRAP3-18-deficient mice (KO) and wild-type mice (WT). Densitometric quantification of the Erdr1 protein expression (data in the left panel) is shown in the right panel. n = 3. * *p* < 0.05.

GTRAP3-18 mRNA and protein are highly expressed in the mouse hippocampus [21,22]. Based on these reports, we utilized the hippocampus of GTRAP3-18-deficient mice to identify genes regulating GTRAP3-18 expression. Microarray gene expression analysis was performed on total RNA isolated from the hippocampus of GTRAP3-18-deficient and wild-type mice (n = 3). The fold change and the significance (*p* < 0.05) of 41,345 genes is shown in a volcano plot (Figure 1a). One hundred and seventy-two genes and 154 genes were significantly up and downregulated, respectively. Genes with greater than 2-fold change and less than 0.5-fold change are shown in Figure 1b. Remarkably, *Erdr1* exhibited the significant change in abundance and was increased 3.22-fold in the hippocampus of GTRAP3-18-deficient mice. This substantial upregulation of Erdr1 in the hippocampus of GTRAP3-18-deficient mice was confirmed through quantitative real-time PCR analysis, n = 5 (Figure 1c) and Western blotting analysis, n = 13 (Figure 1d), respectively. Given the previous reports of Erdr1 expression in the mouse brain [18,20], we further investigated Erdr1 gene and protein expression in the brains of GTRAP3-18-deficient mice. The results revealed upregulation of both *Erdr1* gene, *p* < 0.05 (Figure 1e) and protein expression, *p* < 0.05 (Figure 1f) in the brains of GTRAP3-18-deficient mice. These findings suggest a possible role of Erdr1 in the expression of GTRAP3-18.

### 3.2. Regulation of GTRAP3-18 by Erdr1

**Figure 2:**
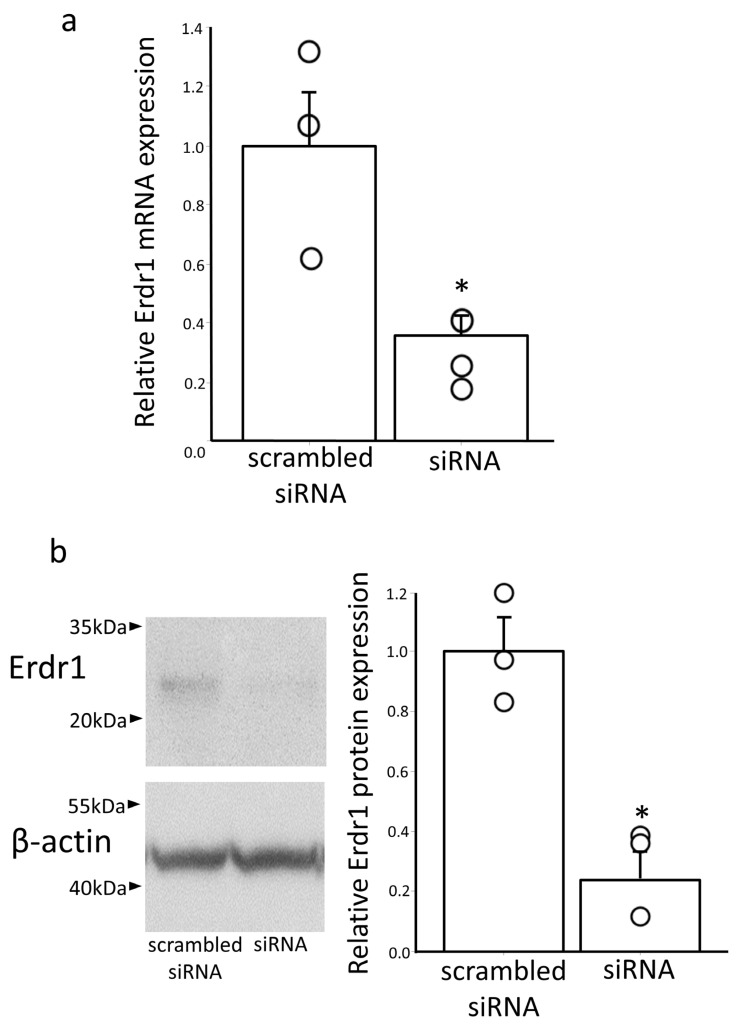
Decreased *Erdr1* gene and protein expression by transfection with a specific siRNA targeting Erdr1. (**a**) Quantitative real-time PCR analysis of Erdr1 levels in Neuro2a cells transfected with Erdr1 siRNA (siRNA) compared to those in cells transfected with scrambled siRNA as a negative control. Data are shown as the ratio of Erdr1 to GAPDH mRNA expression. n = 3. * *p* < 0.05. (**b**) Western blotting analysis of Erdr1 levels in Neuro2a cells transfected with Erdr1 siRNA (siRNA) compared to those in the negative control cells (scrambled siRNA). Quantification of the Erdr1 protein expression (data in the left panel) by densitometry is shown in the right panel. n = 3. * *p* < 0.05.

To assess the potential influence of Erdr1 on GTRAP3-18 expression, we performed a knockdown experiment in the mouse neuroblastoma cell line Neuro2a, employing a specific siRNA targeting *Erdr1* (Table 1). Successful knockdowns of *Erdr1* gene expression (n = 3) and protein expression (n = 3) were achieved, demonstrating reductions exceeding 50%, *p* < 0.05 and 70%, *p* < 0.05, respectively (Figure 2a,b).

**Figure 3:**
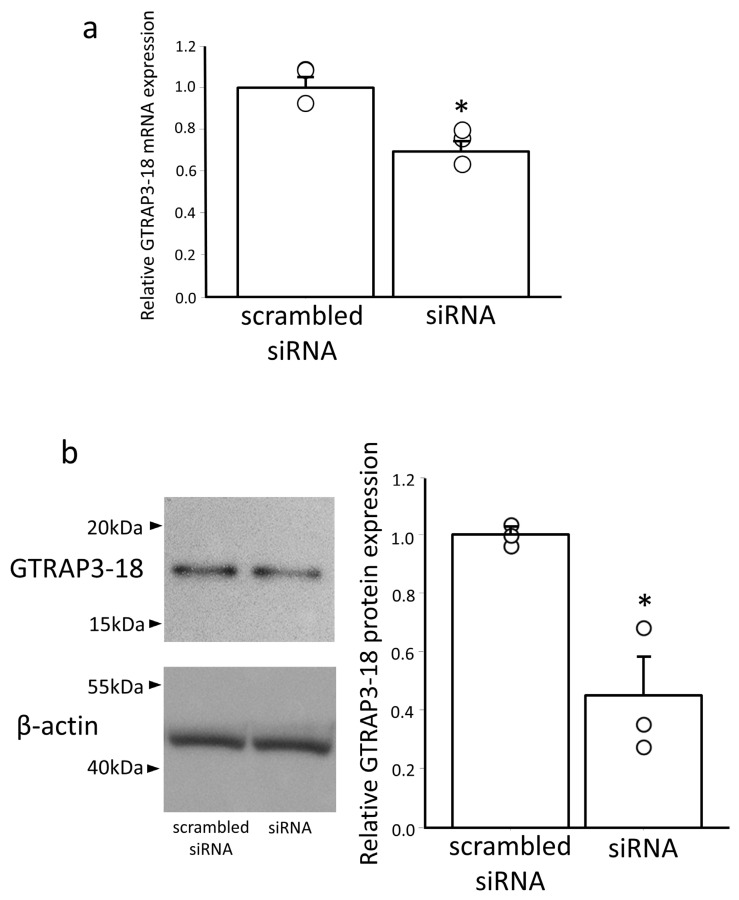
*GTRAP3-18* gene and protein expression were decreased by the knockdown of Erdr1 in Neuro2a cells. (**a**) Quantitative real-time PCR analysis of GTRAP3-18 levels in Neuro2a cells transfected with Erdr1 siRNA (siRNA) compared to those in cells transfected with scrambled siRNA as a negative control. Data are shown as the ratio of GTRAP3-18 to GAPDH mRNA expression. n = 3. * *p* < 0.05. (**b**) Western blotting analysis of GTRAP3-18 levels in Neuro2a cells transfected with Erdr1 siRNA (siRNA) compared to those in the negative control cells (scrambled siRNA). Densitometric quantification of the GTRAP3-18 protein expression (data in the left panel) is shown in the right panel. n = 3. * *p* < 0.05.

Subsequent analysis of the cells transfected with Erdr1 siRNA revealed a significant decrease in both *GTRAP3-18* gene expression (by approximately 30%, *p* < 0.05) and the protein expression (by approximately 50%, *p* < 0.05) compared to the cells transfected with scrambled siRNA, which served as a negative control (Figure 3a,b). These findings indicate that the expression of GTRAP3-18 is regulated by Erdr1.

### 3.3. Effect of Erdr1 on EAAC1 Expression

**Figure 4:**
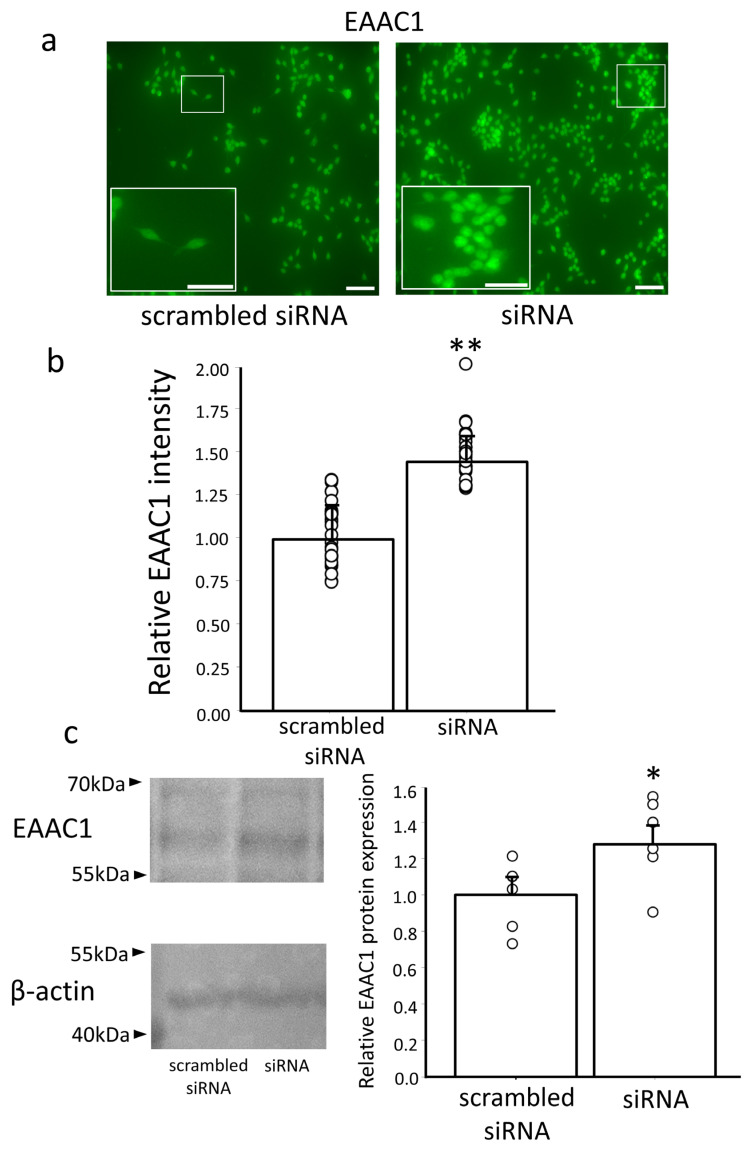
Increased *EAAC1* gene and protein expression in Neuro2a cells with Erdr1 siRNA knockdown. (**a**) Confocal images showing the effect of transfection with a specific siRNA targeting Erdr1 (siRNA) on the intensity of EAAC1 expression in Neuro2a cells compared with that in the negative control cells (scrambled siRNA). Scale bar, 100 μm. Insets in the panels are enlarged images corresponding to the box highlighted on the full images. Scale bar, 20 μm. (**b**) Quantification of relative EAAC1 densities in Neuro2a cells is shown. n = 5; 5–6 areas were measured in each sample. ** *p* < 0.01. (**c**) Protein expression of EAAC1 in Neuro2a cells transfected with a specific siRNA targeting Erdr1 (siRNA) and in the negative control cells (scrambled siRNA). Quantification of the EAAC1 protein expression (data in the left panel) by densitometry is shown in the right panel. n = 6. * *p* < 0.05.

In our previous study [15], we demonstrated that downregulation of GTRAP3-18 led to increased expression of EAAC1 and enhanced GSH synthesis. Building on this knowledge, we hypothesized that Erdr1, which may regulate GTRAP3-18, could also have an impact on the expression of EAAC1. To explore this hypothesis, we conducted an immunocytochemical experiment using an antibody against EAAC1 (n = 5) and performed Western blotting analysis on Neuro2a cells following knockdown of Erdr1 using a specific siRNA (n = 6). The results showed that the expression of EAAC1 was significantly increased by 1.47-fold in cells transfected with Erdr1 siRNA compared to cells transfected with scrambled siRNA, *p* < 0.01 (Figure 4a,b). Furthermore, the analysis of cells transfected with Erdr1 siRNA revealed a 1.29-fold increase in the protein level of EAAC1 compared to the negative control transfection, *p* < 0.05 (Figure 4c). These findings provide compelling evidence that knockdown of Erdr1 leads to an upregulation of EAAC1 expression at both the mRNA and protein levels.

### 3.4. Effect of Erdr1 on GSH Levels

**Figure 5:**
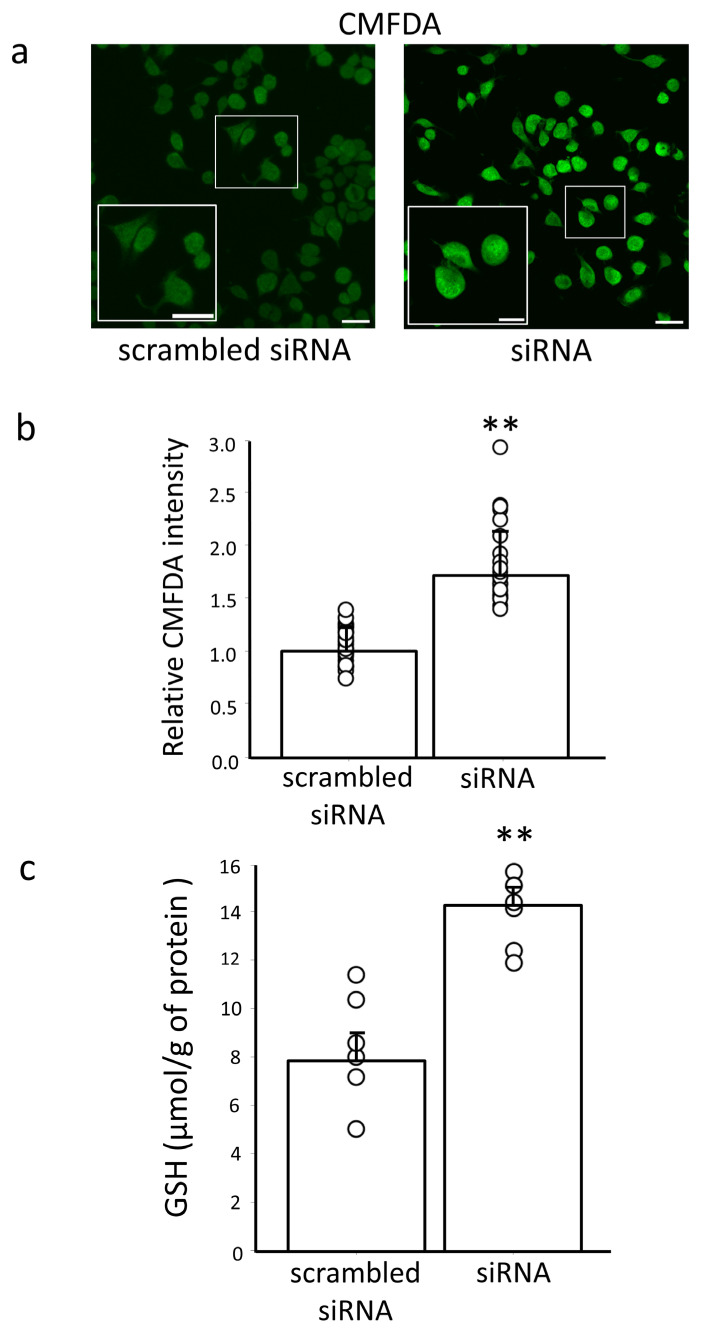
Increased level of GSH by downregulation of Erdr1 in Neuro2a cells. (**a**) Fluorescence microscope images of CMFDA-labeled GSH in Neuro2a cells transfected with Erdr1 siRNA (siRNA) or transfected with scrambled siRNA as a negative control. Scale bar, 100 μm. Insets in the panels are enlarged images corresponding to the box highlighted on the full images. Scale bar, 50 μm. (**b**) The relative fluorescence intensities of CMFDA are shown. n = 4; 5–6 areas were measured in each sample. ** *p* < 0.01. (**c**) Total GSH contents measured by HPLC analysis in both Neuro2a cells transfected with a specific siRNA targeting Erdr1 (siRNA) and the negative controls (scrambled siRNA) are shown as GSH concentrations normalized by the protein concentration of each sample in Neuro2a cells. n = 6. ** *p* < 0.01.

To further investigate the potential regulatory role of Erdr1 on GSH levels, we conducted experiments using Neuro2a cells transfected with Erdr1 siRNA (n = 4). As a marker for GSH, we employed CMFDA, which exhibits fluorescence upon reacting with GSH via a glutathione *S*-transferase-mediated reaction, as previously described [23]. The results, as depicted in Figure 5a,b, clearly demonstrated a significantly higher intensity of CMFDA fluorescence in cells transfected with Erdr1 siRNA compared to the negative control, *p* < 0.01. This observation provides evidence of an elevated GSH content in the cells following downregulation of Erdr1. To validate this finding, we quantitatively assessed the changes in intracellular GSH levels using HPLC (n = 6). Consistent with the fluorescence microscopic analysis results, the GSH levels detected by HPLC analysis were found to be significantly higher in the cells transfected with Erdr1 siRNA compared to the negative control, *p* < 0.01 (Figure 5c). These results collectively indicate that the downregulation of Erdr1 leads to an increase in GSH levels in cultured cells, further supporting the notion that Erdr1 plays a regulatory role in modulating GSH synthesis.

### 3.5. Effect of Erdr1 on GSH Levels in Primary Hippocampal Neurons

**Figure 6:**
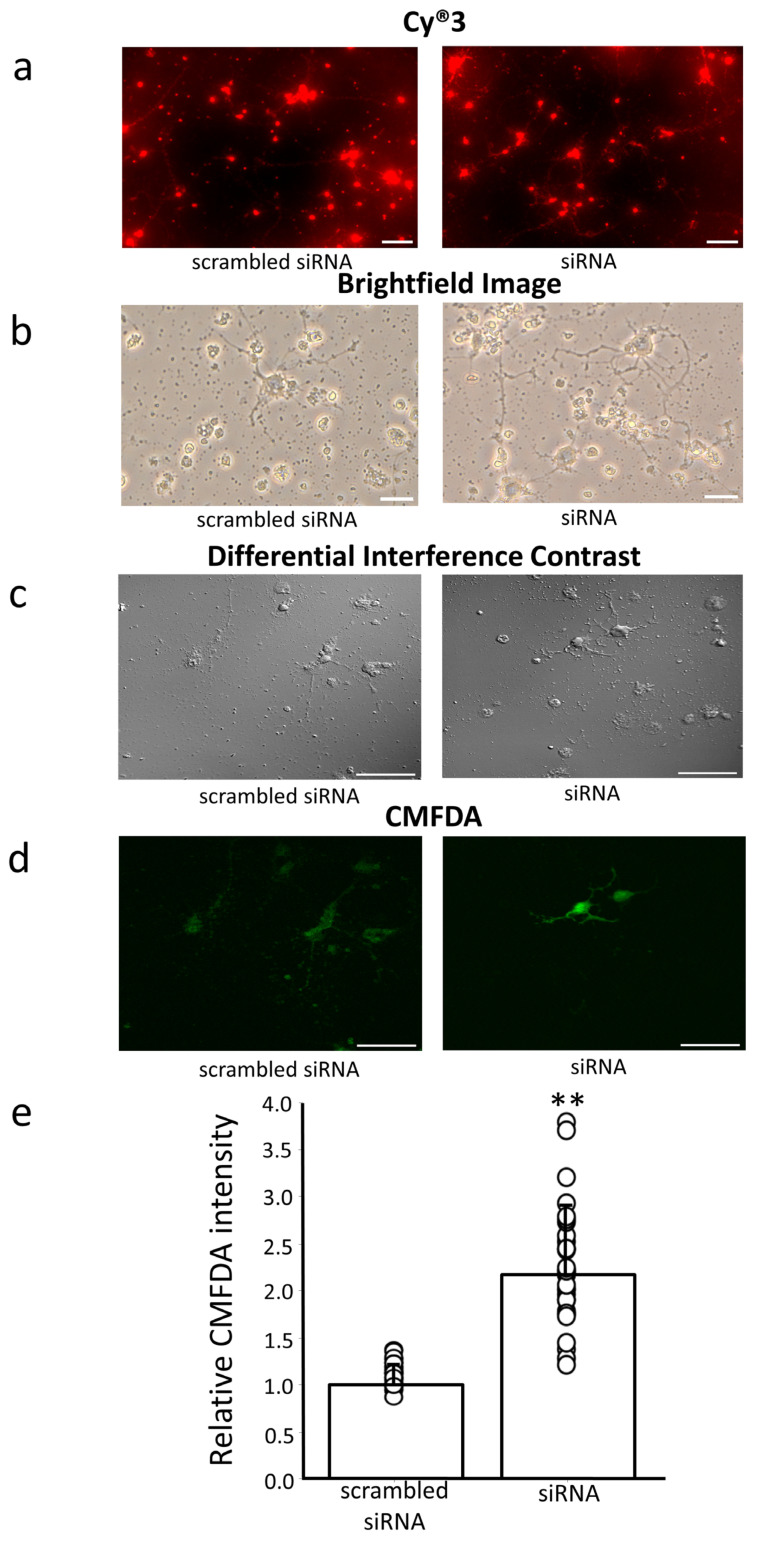
Increased level of GSH by downregulation of Erdr1 in primary mouse hippocampal neurons. (**a**) Fluorescent microscopy images of siRNA specific for Erdr1 labeling with Cy^®^3 dye or a scrambled siRNA labeling with Cy^®^3 dye observed in the primary hippocampal neurons. Scale bar, 100 μm. (**b**) Brightfield images of primary hippocampal neurons transfected with Erdr1 siRNA (siRNA) or transfected with scrambled siRNA. Scale bar, 200 μm. (**c**) Differential interference contrast microscopy of CMFDA-labeled GSH of primary hippocampal neurons transfected with Erdr1 siRNA (siRNA) or transfected with scrambled siRNA. Scale bar, 100 μm. (**d**) Fluorescence microscope images of CMFDA-labeled GSH in primary hippocampal neurons transfected with Erdr1 siRNA (siRNA) or transfected with scrambled siRNA. Scale bar, 100 μm. (**e**) The relative fluorescence intensities of CMFDA are shown. n = 6; 5–6 areas were measured in each sample. ** *p* < 0.01.

To further substantiate the role of Erdr1 in GSH regulation, we utilized primary mouse hippocampal neurons transfected with Erdr1 siRNA (n = 6). Successful siRNA transfection to primary neurons was confirmed by detecting a fluorescent conjugate of Cy^®^3 and siRNA as shown in Figure 6a. By DIV8, primary hippocampal neurons were forming networks as displayed by connections among neurons as shown in Figure 6b and 6c. Subsequently, intracellular GSH levels were assessed by staining with CMFDA and visualized through fluorescent microscopy. A significantly higher intensity of CMFDA was observed in neurons transfected with Erdr1 siRNA compared to the negative control, *p* < 0.01 (Figure 6d,e). This finding supports the notion that downregulation of Erdr1 leads to increased GSH content in neurons.

### 3.6. Knockdown of Erdr1 Protected Neuro2a Cells from H_2_O_2_-Induced Cell Damage

**Figure 7:**
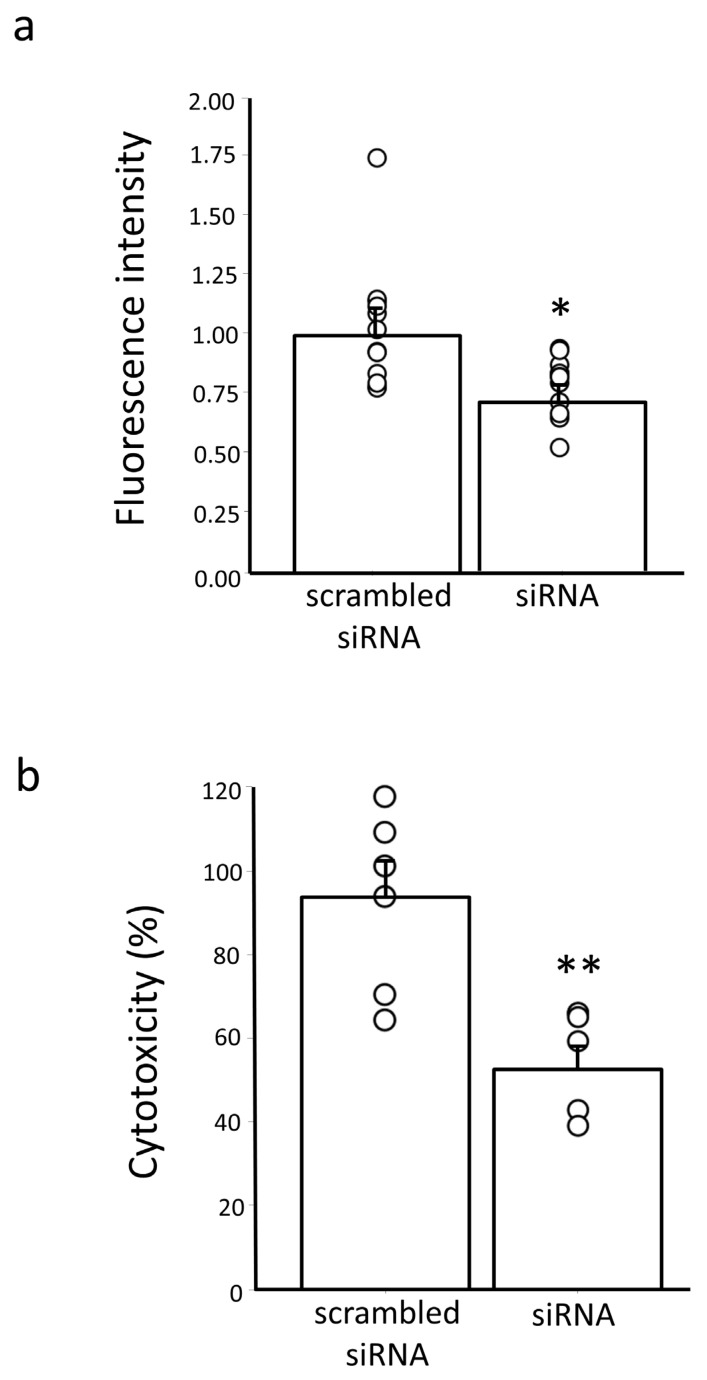
Increased resistance to oxidative stress by knockdown of Erdr1 in Neuro2a cells. (**a**) Fluorescence intensity after 100 μM H_2_O_2_ treatment of Neuro2a cells transfected with Erdr1 siRNA (siRNA) or the scrambled siRNA as a negative control. DCFH-DA, a fluorogenic dye was taken up by the cells. It was then deacetylated by cellular esterase to a non-fluorescent compound, which is later oxidized by ROS into DCF. DCF is highly fluorescent as shown in fluorescence intensity. n = 12. * *p* < 0.05. (**b**) The percentages of cytotoxicity and neuroprotective activities of Erdr1 siRNA-knockdown (siRNA) or negative control (scrambled siRNA) in Neuro2a cells are shown. n = 6. ** *p* < 0.01.

To explore the role of Erdr1 in antioxidant defense, we conducted additional investigations into the impact of Erdr1 on ROS levels using Neuro2a cells transfected with Erdr1 siRNA (n = 12). Following treatment with 100 μM H_2_O_2_, we measured the intracellular ROS levels and found that they were significantly decreased in cells transfected with Erdr1 siRNA compared to the control, *p* < 0.05 (Figure 7a). These findings suggest that the downregulation of Erdr1 expression reduces ROS levels, leading to a protective effect against oxidative stress by increasing GSH levels through enhanced EAAC1 expression in cultured cells. To reinforce the potential neuroprotective activity resulting from the increased expression of EAAC1 and GSH levels following Erdr1 knockdown in Neuro2a cells, we conducted a cytotoxicity assay. Specifically, Neuro2a cells transfected with Erdr1 siRNA were treated with 100 μM H_2_O_2_ for 24 h (n = 6). The results, as shown in Figure 7b, revealed a significant decrease in cell death induced by the 24-h treatment of 100 μM H_2_O_2_ in the Erdr1 siRNA-knockdown Neuro2a cells compared to the control, *p* < 0.01. These findings suggest that the downregulation of Erdr1 exerts a protective effect against oxidative stress by increasing GSH levels in Neuro2a cells.

## 4. Discussion

DNA microarray analysis is a powerful tool for simultaneously analyzing numerous genes at a molecular level [24]. In this study, we investigated gene expression patterns in the hippocampus of GTRAP3-18-deficient mice using DNA microarray analysis to identify genes that might be affected by knockdown of the GTRAP3-18 gene. A total of 172 genes were found to be significantly upregulated by more than 2-fold, and 154 genes were significantly downregulated by less than 0.5-fold.

The observation that GTRAP3-18 mRNA and protein were robustly expressed in the pyramidal cell layer of the mouse hippocampus was in alignment with previous reports [21,22]. The observed downregulation of genes such as *Pmch* in the hippocampus of GTRAP3-18-deficient mice was consistent with the findings of our previous report [25], in which GTRAP3-18-deficient mice exhibited hypophagia, reduced body weight, and altered levels of blood glucose, insulin, and leptin, highlighting the critical role of GTRAP3-18 in the melanocortin pathway. These observations are also in line with the documented phenotypic traits observed in Pmch-deficient mice, which include reduced body weight, loss of body fat, and hypophagia [26,27]. The findings suggest that GTRAP3-18 plays a critical role in regulating metabolic processes and feeding behavior, likely through its involvement in the melanocortin pathway.

Among the identified genes, *Erdr1* appeared to be a highly significant upregulated gene in the hippocampus of GTRAP3-18-deficient mice. The DNA microarray result was consistent with the findings by quantitative real-time PCR and Western blotting analysis, indicating the potential involvement of GTRAP3-18 in the regulation of Erdr1 expression. The present study provides compelling evidence that Erdr1 functions as a key regulator of GTRAP3-18 expression by mediating EAAC1, ultimately influencing the intracellular GSH levels and conferring antioxidant defense in an *in vitro* setting.

Based on the additional results from DNA microarray analysis, quantitative real-time PCR, and Western blotting analysis, Erdr1 showed increased gene and protein expression not only in the hippocampus but also throughout the entire brain of GTRAP3-18-deficient mice. These findings suggest that GTRAP3-18 may be regulated by Erdr1 across the brain, beyond just the hippocampus. Considering the diverse functions of different brain regions, further investigation is warranted to elucidate how Erdr1 operates within each brain region through the modulation of GTRAP3-18 at the molecular level, although the main objective of this study is to elucidate the functional role of Erdr1 within the hippocampus.

Since the upregulation of Erdr1 was observed in the hippocampus of GTRAP3-18-deficient mice, it is assumed that Erdr1, a positive regulator of GTRAP3-18 expression, was compensatorily upregulated in the GTRAP3-18-deficient mice. Conversely, knockdown of Erdr1 results in positive downregulation of GTRAP3-18 expression. Further exploration of the regulatory interactions between Erdr1 and GTRAP3-18, as well as elucidation of their underlying molecular mechanisms, is warranted for future research.

Neuro2a cells derived from mouse neuroblastoma were utilized to investigate the role of Erdr1 in GTRAP3-18 expression. These cells were considered a suitable model due to their neuronal and amoeboid stem cell morphology, and they have been extensively employed for studying neuronal differentiation [28]. Neuro2a cells can be differentiated into neurons within a few days [28,29]. However, neuronal differentiation in cell culture presents a notable limitation: transfection of siRNA into differentiated cell cultures is considerably more challenging compared to undifferentiated ones [30,31]. Considering this limitation, we opted to use undifferentiated Neuro2a cells to investigate the relationship between Erdr1 and GTRAP3-18. We successfully transfected Erdr1 siRNA for the silencing of the *Erdr1* gene using Lipofectamine^®^. Consequently, knockdown of Erdr1 in Neuro2a cells resulted in the suppression of *GTRAP3-18* gene and protein expression. To assess the effects of Erdr1 silencing, GSH levels were measured using both CMFDA [32] for immunostaining and ABD-F for HPLC analysis [33]. In a consistent manner, suppressing the expression of Erdr1 led to an increase in GSH levels, as detected not only by CMFDA fluorescence but also by HPLC analysis in the cells. These findings suggest a regulatory role of Erdr1 in modulating intracellular GSH levels. When combined with our previous study demonstrating that GTRAP3-18 downregulation induces EAAC1 translocation to the plasma membrane and enhances GSH synthesis [15], they further suggest that Erdr1 may influence GTRAP3-18 expression, ultimately leading to elevated GSH levels by promoting EAAC1 expression.

A previous study demonstrated that neurons in the hippocampus of the EAAC1-deficient mice exhibited low GSH and high oxidant levels, leading to susceptibility to oxidative stress [12]. In this study, we employed primary cultures of mouse hippocampal neurons as a model to provide direct evidence of the ability of Erdr1 to increase neuronal GSH content. The use of primary cultures from the hippocampus enabled the examination of the effects of Erdr1 and GSH on antioxidant defense. Using scrambled siRNA as a control and following the same procedures as with Erdr1 siRNA, it was acknowledged that transfection reagents can induce stress in primary cultured cells, often leading to morphological changes even in control groups. Despite this, the results shown in Figure 6 demonstrated that GSH levels were significantly increased following Erdr1 knockdown in primary hippocampal neurons, consistent with the results obtained from Neuro2a cells. These findings indicate that the inhibition of Erdr1 promotes GSH production in neurons.

The critical role of GSH in the CNS, particularly its direct interaction with reactive oxygen species (ROS), is well-established [3]. In the experiment shown in Figure 7, knockdown of Erdr1 not only decreased ROS levels but also conferred cytoprotective effects against oxidative stress induced by H_2_O_2_, although this experiment was conducted using undifferentiated Neuro2a cells instead of primary neuronal cultures for the reasons stated above. Considering that both EAAC1 and GTRAP3-18 are selectively expressed in neurons in the brain, it is plausible to conclude that the antioxidant effects from suppressing Erdr1, which elevates GSH through intrinsic regulation, also provide selective neuroprotection in the CNS.

**Figure 8:**
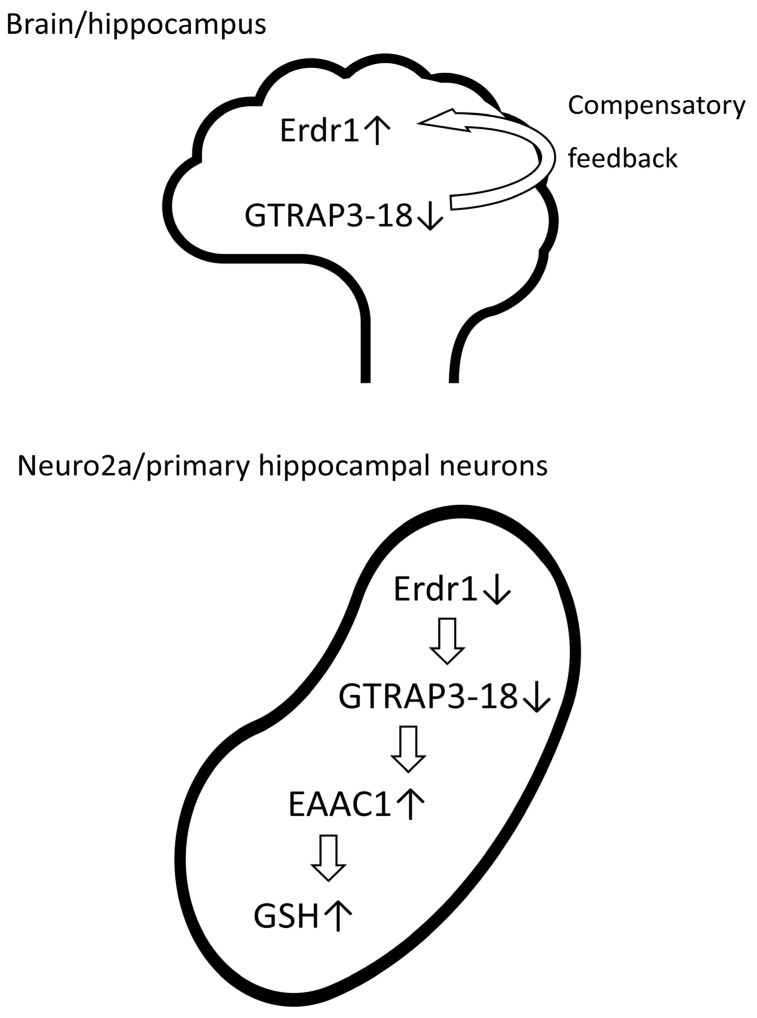
The schematic illustration indicates the relationship between Erdr1, GTRAP3-18, EAAC1, and GSH in the brain/hippocampus and Neuro2a/primary hippocampal neurons. In the brain/hippocampus of GTRAP3-18-deficient mice, GTRAP3-18 may be regulated by Erdr1 and exert compensatory feedback on Erdr1 expression. In Neuro2a/primary hippocampal neurons, knockdown of Erdr1 resulted in a decrease in GTRAP3-18, upregulation of EAAC1 expression, leading to an increase in GSH levels.

In recent years, there has been a growing focus on therapeutic agents for patients with neurodegenerative diseases, with antioxidant therapy emerging as a promising approach against neurodegeneration. Exogenous approaches to increase neuronal GSH levels face challenges due to the limited ability of GSH to cross the blood–brain barrier (BBB) [34]. Given the limited ability of GSH to cross the BBB [34], endogenous mechanisms enhancing neuronal GSH levels may serve as alternative strategies for protecting against neurodegeneration. In summary, the findings of this study highlight the potential role of GTRAP3-18 in gene expression regulation via Erdr1, particularly its influence on the downstream pathways involving EAAC1 and GSH levels as shown in Figure 8. Understanding these molecular mechanisms could have implications for neuroprotection, contribute to a broader understanding of gene regulation in the context of neurological function, and provide a novel therapeutic approach for the treatment of neurodegenerative disorders.

## Figures and Tables

**Table 1:** Erdr1 siRNA information.

	Sense	Antisense
Sequence (5′ → 3′)	GGCCUGACUGCGUACAGAAtt	UUCUGUACGCAGUCAGGCCct
Length	21	21
Percent G/C	52%	57%
Molecular weight	6800	6600
Molecular extinction coefficient	204300	194300
Annealed molecular weight	13400 (40 μg/OD was used to calculate concentration)	

## Data Availability

The authors confirm that the data supporting the findings of this study are available within the article.

## Abbreviations

The following abbreviations are used in this manuscript:

ABD-F, 4-Fluoro-7-sulfamoylbenzofurazan; AD, Alzheimer’s disease; CMFDA, 5-chloromethylfluorescein diacetate; EAAC1, excitatory amino acid carrier 1; EAAT, excitatory amino acid transporter; Erdr1, erythroid differentiation regulator1; GSH, glutathione; GTRAP3-18, glutamate transporter-associated protein 3-18; HPLC, high-performance liquid chromatography; PBS, phosphate buffer saline; PD, Parkinson’s disease; PFA, paraformaldehyde; Pmch, pro-melanin-concentrating hormone.
